# Supplementary material for: Mainly heterosexual, bisexual, or other?: The measurement of sexual minority status and its impact on analytic sample, demographic distribution and health outcomes
Source: PLoS One. 2024 Sep 20;19(9):e0303100. doi: 10.1371/journal.pone.0303100 (PMC11414922; doi:10.1371/journal.pone.0303100)
Supplement: S1 File — (DOCX) [file pone.0303100.s001.docx]

**Supplemental Materials**

Mainly Heterosexual, Bisexual, or Other?: the measurement of sexual minority status and its impact on analytic sample, demographic distribution and health outcomes

Evangeline Tabor, Dylan Kneale, Praveetha Patalay

Table of Contents

[Variable Overlap 3](#_Toc164100699)

[Table S1: Attraction by sexual identity in adolecents 3](#_Toc164100700)

[Table S2: Sexual identity by attraction in adolescents 4](#_Toc164100701)

[Table S3: Attraction by sexual identity in older adults 5](#_Toc164100702)

[Table S4: Experience by sexual identity in older adults 5](#_Toc164100703)

[Table S5: Sexual identity by attraction in older adults 6](#_Toc164100704)

[Table S6: Experience by attraction in older adults 6](#_Toc164100705)

[Table S7: Sexual identity by experience in older adults 7](#_Toc164100706)

[Table S8: Attraction by experience in older adults 7](#_Toc164100707)

[Supplementary Results 8](#_Toc164100708)

[Table S9: Demographic characteristics by sexual identity in adolescents 8](#_Toc164100709)

[Table S10: Demographic characteristics by sexual attraction in adolescents 9](#_Toc164100710)

[Table S11: Demographic characteristics by sexual identity in older adults 11](#_Toc164100711)

[Table S12: Demographic characteristics by sexual attraction in older adults 13](#_Toc164100712)

[Table S13: Demographic characteristics by sexual experience in older adults 15](#_Toc164100713)

[Table S14: Health outcomes by sexual identity in adolescents 17](#_Toc164100714)

[Table S15: Health outcomes by sexual attraction in adolescents 18](#_Toc164100715)

[Table S16: Health outcomes by sexual identity in older adults 19](#_Toc164100716)

[Table S17: Health outcomes by sexual attraction in older adults 20](#_Toc164100717)

[Table S18: Health outcomes by sexual experience in older adults 21](#_Toc164100718)

[Table S19: Health outcomes by sexual identity recoding strategy in older adults 22](#_Toc164100719)

[Table S20: Health outcomes by sexual attraction recoding strategy in older adults 22](#_Toc164100720)

[Table S21: Health outcomes by sexual experience recoding strategy in older adults 23](#_Toc164100721)

[Table S22: Health outcomes by combined dimension recoding strategy in older adults 23](#_Toc164100722)

[Table S23: Health outcomes by sexual identity recoding strategy in adolescents 24](#_Toc164100723)

[Table S23: Health outcomes by sexual attraction recoding strategy in adolescents 24](#_Toc164100724)

[Table S24: Health outcomes by combined dimension recoding strategy in adolescents 25](#_Toc164100725)

# Variable Overlap

## Table S1: Attraction by sexual identity in adolecents

|  | **Completely heterosexual** | **Mainly heterosexual** | **Bisexual** | **Mainly Gay or Lesbian** | **Completely Gay or Lesbian** | **Other** | **Do Not Know** | **Prefer Not to Say** | **Total (%)** |
| --- | --- | --- | --- | --- | --- | --- | --- | --- | --- |
|  | N= 7,888 | N= 1,101 | N= 656 | N= 90 | N= 160 | N= 157 | N= 16 | N = 35 | N= 10,103 |
| **Attraction** |  |  |  |  |  |  |  |  |  |
| **Only to opposite sex (%)** | 7335  (92.99) | 283  (25.70) | 5  (0.76) | 0  (0) | <5  (<0.50) | 27  (17.20) | <5  (<20.00) | <5  (<7.50) | 7656  (75.78) |
| **More often to opposite sex (%)** | 361  (4.58) | 757  (68.76) | 243  (37.04) | <5  (<2.50) | 0  (0) | 17  (10.83) | <5  (<15.00) | <5  (<5.00) | 1382  (13.68) |
| **About equally (%)** | 6  (0.08) | 24  (2.18) | 316  (48.17) | 7  (7.78) | 0  (0) | 30  (19.11) | <5  (<7.50) | <5  (<5.00) | 385  (3.81) |
| **More often to same sex (%)** | 0  (0) | <5  (<0.50) | 72  (10.98) | 74  (82.22) | 33  (20.63) | 13  (8.28) | 0  (0) | 0  (0) | 194  (1.92) |
| **Only ever to same sex (%)** | <5  (<0.50) | <5  (<0.50) | <5  (<0.50) | 7  (7.78) | 121  (75.63) | <5  (<2.50) | 0  (0) | (0) | 137  (1.36) |
| **I have never felt sexual attraction(%)** | 160  (2.03) | 32  (2.91) | 17  (2.59) | <5  (<2.50) | <5  (<2.50) | 64  (40.76) | 7  (43.75) | 6  (17.14) | 291  (2.88) |
| **Do not know (%)** | <5  (<0.50) | 0  (0) | 0  (0) | 0  (0) | <5  (<2.50) | <5  (<2.50) | <5  (<15.00) | <5  (<7.50) | 11  (0.11) |
| **I do not wish to answer (%)** | 18  (0.23) | <5  (<0.50) | <5  (<0.50) | 0  (0) | 0  (0) | <5  (<2.50) | <5  (<7.50) | 23  (65.71) | 46  (0.46) |
| **No answer (%)** | - | - | - | - | - | - | - | - | <5  (<0.50) |

|  | **Only to opposite, never to same** | **More often to opposite, and at least once to a same** | **About equally often to opposite and to opposite** | **More often to same, and at least once to a opposite** | **Only ever to same , never to opposite** | **I have never felt sexually attracted to anyone at all** | **Do not know** | **I do not wish to answer** | **No answer** | **Total** |
| --- | --- | --- | --- | --- | --- | --- | --- | --- | --- | --- |
|  | N=7656 | N=1382 | N=385 | N=194 | N=137 | N=291 | N=11 | N=46 | N=1 | N=10,103 |
| **Completely heterosexual/ straight** | 7335 (95.81) | 361 (26.12) | 6 (1.56) | 0 (0.00) | <5  (<5.00) | 160 (54.98) | <5  (<40.00) | 18 (39.13) | - | 7888 (76.25) |
| **Mainly heterosexual/ straight** | 283 (3.70) | 757 (54.78) | 24 (6.23) | <5  (<2.50) | <5  (<2.50) | 32 (11.00) | 0 (0.00) | <5  (<5.00) | - | 1101 (10.64) |
| **Bisexual** | 5 (0.07) | 243 (17.58) | 316 (82.08) | 72 (37.11) | <5  (<2.50) | 17 (5.84) | 0 (0.00) | <5  (<2.50) | - | 656 (6.34) |
| **Mainly gay or lesbian** | 0 (0.00) | <5  (<0.50) | 7 (1.82) | 74 (38.14) | 7 (5.11) | <5  (<0.50) | 0 (0.00) | 0 (0.00) | - | 90 (0.87) |
| **Completely gay or lesbian** | <5  (<0.50) | 0 (0.00) | 0 (0.00) | 33 (17.01) | 121 (88.32) | <5  (<2.50) | <5  (<10.00) | 0 (0.00) | - | 160 (1.55) |
| **Other** | 27 (0.35) | 17 (1.23) | 30 (7.79) | 13 (6.70) | <5  (<2.50) | 64 (21.99) | <5  (<20.00) | <5  (<2.50) | - | 157 (1.52) |
| **Do not know** | <5  (<0.50) | <5  (<0.50) | <5  (<0.50) | 0 (0.00) | 0 (0.00) | 7 (2.41) | <5  (<20.00) | <5  (<2.50) | - | 16 (0.15) |
| **Prefer not to say** | <5  (<0.50) | <5  (<0.50) | <5  (<0.50) | 0 (0.00) | 0 (0.00) | 6 (2.06) | <5  (<20.00) | 23 (50.00) | - | 35 (0.34) |

## Table S2: Sexual identity by attraction in adolescents

## Table S3: Attraction by sexual identity in older adults

|  | **Heterosexual or Straight** | **Gay or Lesbian** | **Bisexual** | **Other** | **Prefer not to say** | **Missing** | **Total** |
| --- | --- | --- | --- | --- | --- | --- | --- |
| N= | 4513 | 43 | 31 | 27 | 152 | 2364 | 7130 |
| **Experience** |  |  |  |  |  |  |  |
| **Entirely with opposite** | 3443 (76.29) | 0 (0.00) | 8 (25.81) | 17 (62.96) | 89 (58.55) | 2233 (94.46) | 5790 (81.21) |
| **Mostly with opposite, but some experience with same** | 98 (2.17) | <5  (<5.00) | 11 (35.48) | <5  (<7.50) | <5  (<2.50) | 40 (1.69) | 158 (2.22) |
| **Equally with opposite and same** | <5  (<0.50) | <5  (<7.50) | <5  (<10.00) | 0 (0.00) | <5  (<2.50) | 13 (0.55) | 23 (0.32) |
| **Mostly with same, but some experience with opposite** | <5  (<0.50) | 11 (25.58) | <5  (<5.00) | 0 (0.00) | <5  (<2.50) | 13 (0.55) | 27 (0.38) |
| **Entirely with same** | <5  (<0.50) | 16 (37.21) | 0 (0.00) | 0 (0.00) | 0 (0.00) | 13 (0.55) | 33 (0.46) |
| **No sexual experience in lifetime** | 22 (0.49) | 0 (0.00) | 0 (0.00) | 5 (18.52) | 7 (4.61) | 45 (1.90) | 79 (1.11) |
| **Missing** | 944 (20.92) | 9 (20.93) | 8 (25.81) | <5  (<15.00) | 49 (32.24) | 7 (0.30) | 2241 (31.03) |

|  | **Heterosexual or Straight** | **Gay or Lesbian** | **Bisexual** | **Other** | **Prefer not to say** | **Missing** | **Total** |
| --- | --- | --- | --- | --- | --- | --- | --- |
| N= | 4513 | 43 | 31 | 27 | 152 | 2364 | 7130 |
| **Attraction** |  |  |  |  |  |  |  |
| **Entirely with opposite** | 3369 (74.65) | <5  (<2.50) | 7 (22.58) | 15 (55.56) | 83 (54.61) | 2191 (92.68) | 5666 (79.47) |
| **Mostly with opposite, but some experience with same** | 147 (3.26) | <5  (<7.50) | 11 (35.48) | <5  (<10.00) | <5  (<5.00) | 51 (2.16) | 218 (3.06) |
| **Equally with opposite and same** | 22 (0.49) | <5  (<7.50) | <5  (<10.00) | 0 (0.00) | <5  (<5.00) | 25 (1.06) | 57 (0.80) |
| **Mostly with same, but some experience with opposite** | <5  (<0.50) | <5  (<10.00) | <5  (<7.50) | 0 (0.00) | <5  (<2.50) | 10 (0.42) | 19 (0.27) |
| **Entirely with same** | <5  (<0.50) | 22 (51.16) | 0 (0.00) | <5  (<7.50) | 0 (0.00) | 19 (0.80) | 46 (0.65) |
| **No sexual experience in lifetime** | 21 (0.47) | 0 (0.00) | 0 (0.00) | <5  (<15.00) | 11 (7.24) | 51 (2.16) | 87 (1.22) |
| **Missing** | 949 (21.03) | 10 (23.26) | 8 (25.81) | <5  (<15.00) | 49 (32.24) | 17 (0.72) | 1037 (14.54) |

## Table S4: Experience by sexual identity in older adults

## Table S5: Sexual identity by attraction in older adults

|  | **Entirely with opposite** | **Mostly with opposite, but some experience with same** | **Equally with opposite and same** | **Mostly with same, but some experience with opposite** | **Entirely with same** | **No sexual experience in lifetime** | **Missing** | **Total** |
| --- | --- | --- | --- | --- | --- | --- | --- | --- |
| N= | 5666 | 218 | 57 | 19 | 46 | 87 | 1037 | 7130 |
| **Sexual Identity** |  |  |  |  |  |  |  |  |
| **Heterosexual or Straight** | 3369 (59.46) | 147 (67.43) | 22 (38.60) | <5  (<15.00) | <5  (<7.50) | 21 (24.14) | 949 (91.51) | 4513 (63.30) |
| **Gay or Lesbian** | <5  (<0.50) | <5  (<2.50) | <5  (<7.50) | <5  (<25.00) | 22 (47.83) | 0 (0.00) | 10 (0.96) | 43 (0.60) |
| **Bisexual** | 7 (0.12) | 11 (5.05) | <5  (<7.50) | <5  (<15.00) | 0 (0.00) | 0 (0.00) | 8 (0.77) | 31 (0.43) |
| **Other** | 15 (0.26) | <5  (<2.50) | 0 (0.00) | 0 (0.00) | <5  (<5.00) | <5  (<5.00) | <5  (<0.50) | 27 (0.38) |
| **Prefer not to say** | 83 (1.46) | <5  (<2.50) | <5  (<7.50) | <5  (<7.50) | 0 (0.00) | 11 (12.64) | 49 (4.73) | 152 (2.13) |
| **Missing** | 2191 (38.67) | 51 (23.39) | 25 (43.86) | 10 (52.63) | 19 (41.30) | 51 (58.62) | 17 (1.64) | 2364 (33.16) |

## Table S6: Experience by attraction in older adults

|  | **Entirely with opposite** | **Mostly with opposite, but some experience with same** | **Equally with opposite and same** | **Mostly with same, but some experience with opposite** | **Entirely with same** | **No sexual experience in lifetime** | **Schedule not applicable** | **Total** |
| --- | --- | --- | --- | --- | --- | --- | --- | --- |
| N= | 5666 | 218 | 57 | 19 | 46 | 87 | 1037 | 7130 |
| **Sexual experience** |  |  |  |  |  |  |  |  |
| **Entirely with opposite** | 5561 (98.15) | 140 (64.22) | 36 (63.16) | <5  (<15.00) | <5  (<5.00) | 27 (31.03) | 22 (2.12) | 5790 (81.21) |
| **Mostly with opposite, but some experience with same** | 78 (1.38) | 72 (33.03) | 5 (8.77) | <5  (<7.50) | 0 (0.00) | <5  (<2.50) | 0 (0.00) | 158 (2.22) |
| **Equally with opposite and same** | <5  (<0.50) | <5  (<2.50) | 11 (19.30) | 3 (15.79) | <5  (<5.00) | <5  (<2.50) | 0 (0.00) | 23 (0.32) |
| **Mostly with same, but some experience with opposite** | 0 (0.00) | 0 (0.00) | <5  (<2.50) | 11 (57.89) | 13 (28.26) | <5  (<2.50) | <5  (<2.50) | 27 (0.38) |
| **Entirely with same** | <5  (<0.50) | 0 (0.00) | 0 (0.00) | <5  (<15.00) | 27 (58.70) | <5  (<2.50) | 0 (0.00) | 33 (0.46) |
| **No sexual experience in lifetime** | 14 (0.25) | <5  (<2.50) | <5  (<5.00) | 0 (0.00) | <5  (<5.00) | 54 (62.07) | 5 (0.48) | 79 (1.11) |
| **Not answered** | 8 (0.14) | 0 (0.00) | <5  (<5.00) | 0 (0.00) | 0 (0.00) | <5  (<2.50) | 1009 (97.30) | 1020 (14.31) |

|  | **Entirely with opposite** | **Mostly with opposite, but some experience with same** | **Equally with opposite and same** | **Mostly with same, but some experience with opposite** | **Entirely with same** | **No sexual experience in lifetime** | **Missing** | **Total** |
| --- | --- | --- | --- | --- | --- | --- | --- | --- |
| N= | 5790 | 158 | 23 | 27 | 33 | 79 | 1020 | 7130 |
| **Sexual Identity** |  |  |  |  |  |  |  |  |
| **Heterosexual or Straight** | 3443 (74.20) | 98 (71.53) | <5  (<7.50) | <5  (<5.00) | <5  (<20.00 | 22 (38.60) | 32 (37.21) | 4513 (63.30) |
| **Gay or Lesbian** | 0 (0.00) | <5  (<5.00) | <5  (<20.00) | 11 (55.00) | 16 (61.54) | 0 (0.00) | 0 (0.00) | 43 (0.60) |
| **Bisexual** | 8 (0.17) | 11 (8.03) | <5  (<20.00 | <5  (<5.00) | 0 (0.00) | 0 (0.00) | 0 (0.00) | 31 (0.43) |
| **Other** | 17 (0.37) | <5  (<2.50) | 0 (0.00) | 0 (0.00) | 0 (0.00) | 5 (8.77) | <5  (<2.50) | 27 (0.38) |
| **Prefer not to say** | 89 (1.92) | <5  (<2.50) | <5  (<20.00 | <5  (<5.00) | 0 (0.00) | 7 (12.28) | 9 (10.47) | 152 (2.13) |
| **Missing** | 2233 (38.57) | 40 (25.32) | 13 (56.52) | 13 (48.15) | 13 (39.39) | 45 (56.96) | 7 (0.69) | 2364 (33.16) |

## Table S7: Sexual identity by experience in older adults

## Table S8: Attraction by experience in older adults

|  | **Entirely with opposite** | **Mostly with opposite, but some experience with same** | **Equally with opposite and same** | **Mostly with same, but some experience with opposite** | **Entirely with same** | **No sexual experience in lifetime** | **Missing** | **Total** |
| --- | --- | --- | --- | --- | --- | --- | --- | --- |
| N= | 5790 | 158 | 23 | 27 | 33 | 79 | 1020 | 7130 |
| **Sexual Attraction** |  |  |  |  |  |  |  |  |
| **Entirely with opposite** | 5561 (96.04) | 78 (49.37) | <5  (<10.00) | 0 (0.00) | 3 (9.09) | 14 (17.72) | 8 (0.78) | 5666 (79.47) |
| **Mostly with opposite, but some experience with same** | 140 (2.42) | 72 (45.57) | <5  (<20.00) | 0 (0.00) | 0 (0.00) | <5  (<5.00) | 0 (0.00) | 218 (3.06) |
| **Equally with opposite and same** | 36 (0.62) | 5 (3.16) | 11 (47.83) | <5  (<5.00) | 0 (0.00) | <5  (<5.00) | <5  (<0.50) | 57 (0.80) |
| **Mostly with same, but some experience with opposite** | <5  (<0.50) | <5  (<2.50) | <5  (<15.00) | 11 (40.74) | <5  (<5.00) | 0 (0.00) | 0 (0.00) | 19 (0.27) |
| **Entirely with same** | <5  (<0.50) | 0 (0.00) | <5  (<5.00) | 13 (48.15) | 27 (81.82) | <5  (<5.00) | 0 (0.00) | 46 (0.65) |
| **No sexual experience in lifetime** | 27 (0.47) | <5  (<2.50) | <5  (<5.00) | <5  (<5.00) | <5  (<5.00) | 54 (68.35) | <5  (<0.50) | 87 (1.22) |
| **Missing** | 22 (0.38) | 0 (0.00) | 0 (0.00) | <5  (<5.00) | 0 (0.00) | 5 (6.33) | 1009 (98.92) | 1037 (14.54) |

# Supplementary Results

## Table S9: Demographic characteristics by sexual identity in adolescents

|  | **Completely heterosexual** | **Mainly heterosexual** | **Bisexual** | **Mainly Gay or Lesbian** | **Completely Gay or Lesbian** | **Other** | **Do Not Know** | **Prefer Not to Say** | **Total** |
| --- | --- | --- | --- | --- | --- | --- | --- | --- | --- |
|  | N= 7,888 | N= 1,101 | N= 656 | N= 90 | N= 160 | N= 157 | N= 16 | N = 35 | N= 10,103 |
| **Gender Identity (Four category)** |  |  |  |  |  |  |  |  |  |
| Male | 4154 (52.66) | 399 (36.24) | 159 (24.24) | 33 (36.67) | 86 (53.75) | 40 (25.48) | 5 (31.25) | 8 (22.86) | 4884 (48.34) |
| Female | 3719 (47.15) | 691 (62.76) | 472 (71.95) | 47 (52.22) | 63 (39.38) | 74 (47.13) | 10 (62.50) | 15 (42.86) | 5091 (50.39) |
| Non-binary/Other | 0 (0.00) | <5 (<0.50) | 14 (2.13) | 7 (7.78) | 10 (6.25) | 25 (15.92) | 0 (0.00) | 0 (0.00) | 58 (0.57) |
| Don’t know / PNS | 9 (0.11) | 5 (0.45) | 11 (1.68) | <5 (<5.00) | <5 (<2.50) | 18 (11.46) | <5 (<7.50) | 11 (31.43) | 59 (0.58) |
| Missing | 6 (0.08) | <5 (<2.50) | 0 (0.00) | 0 (0.00) | 0 (0.00) | 0 (0.00) | 0 (0.00) | <5 (<5.00) | 11 (0.11 |
| **Ethnicity** |  |  |  |  |  |  |  |  |  |
| White | 6233 (79.02) | 929 (84.38) | 587 (89.48) | 81 (90.00) | 146 (91.25) | 129 (82.17) | 11 (68.75) | 24 (68.57) | 8,140 (80.59) |
| **OECD Equivalised income quintiles** |  |  |  |  |  |  |  |  |  |
| Lower quantile | 1325 (16.80) | 108 (9.81) | 83 (12.65) | 8 (8.89) | 25 (15.63) | 26 (16.56) | <5 (<25.00) | 12 (34.29) | 1,591 (15.75) |
| Second quantile | 1236 (15.67) | 145 (13.17) | 100 (15.24) | 12 (13.33) | 36 (22.50) | 31 (19.75) | 5 (31.25) | 9 (25.71) | 1,576 (15.60) |
| Third quantile | 1568 (19.88) | 211 (19.16) | 128 (19.51) | 15 (16.67) | 26 (16.25) | 30 (19.11) | 0 (0.00) | 5 (14.29) | 1,988 (19.68) |
| Fourth quantile | 1831 (23.21) | 271 (24.61) | 147 (22.41) | 26 (28.89) | 31 (19.38) | 31 (19.75) | <5 (<15.50) | 6 (17.14) | 2,346 (23.22) |
| Highest quantile | 1750 (22.19) | 341 (30.97) | 183 (27.90) | 28 (31.11) | 38 (23.75) | 38 (24.20) | <5 (<25.00) | <5 (<5.00) | 2,387 (23.63) |
| Missing | 178 (2.26) | 25 (2.27) | 15 (2.29) | <5 (<2.50) | <5 (<2.50) | <5 (<2.50) | <5 (<7.50) | <5 (<7.50) | 215 (2.13 |
| **Highest parent/guardian qualification (NVQ Level)** |  |  |  |  |  |  |  |  |  |
| NVQ Level 1 | 327 (4.15) | 21 (1.91) | 23 (3.51) | <5 (<2.50) | 6 (3.75) | <5 (<2.50) | <5 (<7.50) | <5 (<15.00) | 388 (3.84) |
| NVQ Level 2 | 1384 (17.55) | 164 (14.90) | 97 (14.79) | 18 (20.00) | 33 (20.63) | 27 (17.20) | <5 (<20.00) | 9 (25.71) | 1,742 (17.42) |
| NVQ Level 3 | 1135 (14.39) | 115 (10.45) | 81 (12.35) | 8 (8.89) | 27 (16.88) | 20 (12.74) | <5 (<25.00) | <5 (<7.50) | 1,392 (13.78) |
| NVQ Level 4 | 2863 (36.30) | 445 (40.42) | 257 (39.18) | 38 (42.22) | 49 (30.63) | 61 (38.85) | <5 (<20.00) | 7 (20.00) | 3,725 (36.87) |
| NVQ Level 5 | 1403 (17.79) | 286 (25.98) | 143 (21.80) | 22 (24.44) | 27 (16.88) | 30 (19.11) | <5 (<15.50) | 0 (0.00) | 1,913 (18.93) |
| None | 371 (4.70) | 27 (2.45) | 18 (2.74) | <5 (<2.50) | 10 (6.25) | 11 (7.01) | <5 (<15.50) | 5 (14.29) | 454 (4.49) |
| Overseas/Other | 166 (2.10) | 12 (1.09) | 16 (2.44) | 0 (0.00) | <5 (<2.50) | <5 (<2.50) | 0 (0.00) | <5 (<10.00) | 206 (2.04) |
| Missing | 239 (3.03) | 31 (2.82) | 21 (3.20) | <5 (<2.50) | <5 (<2.50) | <5 (<2.50) | <5 (<7.50) | 5 (14.29) | 283 (2.80) |
| **ONS Urban/Rural Classification 2005** |  |  |  |  |  |  |  |  |  |
| Rural | 1783 (22.60) | 298 (27.07) | 152 (23.17) | 14 (15.56) | 21 (13.13) | 39 (24.84) | 0 (0.00) | <5 (<10.00) | 2,310 (22.86) |
| Urban | 5841 (74.05) | 768 (69.75) | 483 (73.63) | 73 (81.11) | 135 (84.38) | 115 (73.25) | 15 (93.75) | 29 (82.86) | 7,459 (73.83) |
| Missing | 264 (3.35) | 35 (3.18) | 21 (3.20) | <5 (<5.00) | <5 (<2.50) | <5 (<2.50) | <5 (<7.50) | <5 (<10.00) | 334 (3.31) |

## Table S10: Demographic characteristics by sexual attraction in adolescents

|  | **Only to opposite** | **More often to opposite** | **About equally** | **More often to same** | **Only to same** | **Never sexually attracted** | **Do not know** | **I do not wish to answer** | **Missing** | **Total** |
| --- | --- | --- | --- | --- | --- | --- | --- | --- | --- | --- |
| **N=10,345)** | N=7,656 | N=1,382 | N=385 | N=194 | N=137 | N=291 | N=11 | N=46 | N=1 | N=10,103 |
| **Gender Identity (Four category)** |  |  |  |  |  |  |  |  |  |  |
| Male | 4155 (54.27) | 399 (28.87) | 81 (21.04) | 67 (34.54) | 75 (54.74) | 96 (32.99) | 2 (18.18) | 9 (19.57) | - | 4884 (48.34) |
| Female | 3476 (45.40) | 969 (70.12) | 280 (72.73) | 108 (55.67) | 51 (37.23) | 174 (59.79) | 5 (45.45) | 28 (60.87) | - | 5091 (50.39) |
| Non-binary/Other | 2 (0.03) | 8 (0.58) | 15 (3.90) | 14 (7.22) | 8 (5.84) | 10 (3.44) | 0 (0.00) | 0 (0.00) | - | 58 (0.57) |
| Don’t know / PNS | 15 (0.20) | <5 (<0.50) | 9 (2.34) | 5 (2.58) | <5 (<2.50) | 11 (3.78) | <5 (<40.00) | 8 (17.39) | - | 59 (0.58) |
| Missing | 8 (0.10) | <5 (<0.50) | 0 (0.00) | 0 (0.00) | 0 (0.00) | 0 (0.00) | 0 (0.00) | <5 (<2.50) | - | 11 (0.11 |
| **Ethnicity** |  |  |  |  |  |  |  |  |  |  |
| White | 6072 (79.31) | 1204 (87.12) | 343 (89.09) | 167 (86.08) | 127 (92.70) | 190 (65.29) | 6 (54.55) | 30 (65.22) | - | 8,140 (80.59) |
| **OECD Equivalised income quintiles** |  |  |  |  |  |  |  |  |  |  |
| Lower quantile | 1265 (16.52) | 126 (9.12) | 52 (13.51) | 17 (8.76) | 25 (18.25) | 84 (28.87) | <5 (<40.00) | 18 (39.13) | - | 1,591 (15.75) |
| Second quantile | 1196 (15.62) | 191 (13.82) | 66 (17.14) | 25 (12.89) | 33 (24.09) | 53 (18.21) | <5 (<30.00) | 7 (15.22) | - | 1,576 (15.60) |
| Third quantile | 1512 (19.75) | 288 (20.84) | 69 (17.92) | 44 (22.68) | 18 (13.14) | 42 (14.43) | <5 (<10.00) | 9 (19.57) | - | 1,988 (19.68) |
| Fourth quantile | 1781 (23.26) | 355 (25.69) | 76 (19.74) | 48 (24.74) | 27 (19.71) | 49 (16.84) | <5 (<20.00) | 7 (15.22) | - | 2,346 (23.22) |
| Highest quantile | 1732 (22.62) | 391 (28.29) | 108 (28.05) | 58 (29.90) | 31 (22.63) | 57 (19.59) | <5 (<10.00) | <5 (<10.00) | - | 2,387 (23.63) |
| Missing | 170 (2.22) | 31 (2.24) | 14 (3.64) | 2 (1.03) | <5 (<2.50) | 6 (2.06) | 0 (0.00) | <5 (<2.50) | - | 215 (2.13 |
| **Highest parent/guardian qualification (NVQ Level)** |  |  |  |  |  |  |  |  |  |  |
| NVQ Level 1 | 305 (3.98) | 34 (2.46) | 12 (3.12) | 5 (2.58) | 5 (3.65) | 21 (7.22) | 2 (18.18) | <5 (<7.50) | - | 388 (3.84) |
| NVQ Level 2 | 1341 (17.52) | 208 (15.05) | 56 (14.55) | 36 (18.56) | 25 (18.25) | 55 (18.90) | <5 (<30.00) | 11 (23.91) | - | 1,742 (17.42) |
| NVQ Level 3 | 1091 (14.25) | 173 (12.52) | 45 (11.69) | 24 (12.37) | 19 (13.87) | 36 (12.37) | 0 (0.00) | 5 (<10.00) | - | 1,392 (13.78) |
| NVQ Level 4 | 2774 (36.23) | 561 (40.59) | 143 (37.14) | 79 (40.72) | 45 (32.85) | 107 (36.77) | <5 (<30.00) | 11 (23.91) | - | 3,725 (36.87) |
| NVQ Level 5 | 1394 (18.21) | 322 (23.30) | 90 (23.38) | 42 (21.65) | 26 (18.98) | 36 (12.37) | 0 (0.00) | <5 (<5.00) | - | 1,913 (18.93) |
| None | 353 (4.61) | 31 (2.24) | 14 (3.64) | <5 (<2.50) | 10 (7.30) | 23 (7.90) | <5 (<30.00) | 8 (17.39) | - | 454 (4.49) |
| Overseas/Other | 170 (2.22) | 9 (0.65) | 10 (2.60) | <5 (<2.50) | <5 (<5.00) | 5 (1.72) | 0 (0.00) | <5 (<7.50) | - | 206 (2.04) |
| Missing | 228 (2.98) | 44 (3.18) | 15 (3.90) | <5 (<2.50) | <5 (<2.50) | 8 (2.75) | 0 (0.00) | 5 (<10.00) | - | 283 (2.80) |
| **ONS Urban/Rural Classification 2005** |  |  |  |  |  |  |  |  |  |  |
| Rural | 1747 (22.82) | 372 (26.92) | 80 (20.78) | 32 (16.49) | 22 (16.06) | 51 (17.53) | <5 (<10.00) | 5 (<10.00) | - | 2,310 (22.86) |
| Urban | 5653 (73.84) | 967 (69.97) | 289 (75.06) | 158 (81.44) | 111 (81.02) | 231 (79.38) | 10 (90.91) | 40 (86.96) | - | 7,459 (73.83) |
| Missing | 256 (3.34) | 43 (3.11) | 16 (4.16) | <5 (<0.50) | 5 (<5.00) | 9 (3.09) | 0 (0.00) | <5 (<5.00) | - | 334 (3.31) |

## Table S11: Demographic characteristics by sexual identity in older adults

|  | **Heterosexual or Straight** | **Gay or Lesbian** | **Bisexual** | **Other** | **Prefer Not to Say** | **Missing** | **Total** |
| --- | --- | --- | --- | --- | --- | --- | --- |
| N= | 4513 | 43 | 31 | 27 | 152 | 2364 | 7130 |
| **Sex** |  |  |  |  |  |  |  |
| Male | 2009 (44.52) | 26 (60.47) | 20 (64.52) | 12 (44.44) | 63 (41.45) | 1054 (44.59) | 3184 (44.66) |
| Female | 2504 (55.48) | 17 (39.53) | 11 (35.48) | 15 (55.56) | 89 (58.55) | 1310 (55.41) | 3946 (55.34) |
| **Age bands** |  |  |  |  |  |  |  |
| 50-59 | 584 (12.94) | 8 (18.60) | <5 (<15.00) | 0 (0.00) | 10 (6.58) | 310 (13.11) | 916 (12.85) |
| 60-69 | 2011 (44.56) | 19 (44.19) | 14 (45.16) | 9 (33.33) | 48 (31.58) | 723 (30.58) | 2824 (39.61) |
| 70-79 | 1387 (30.73) | 16 (37.21) | 13 (41.94) | 7 (25.93) | 52 (34.21) | 758 (32.06) | 2233 (31.32) |
| 80-89 | 490 (10.86) | 0 (0.00) | 0 (0.00) | 9 (33.33) | 40 (26.32 ) | 476 (20.14) | 1015 (14.24) |
| 90+ | 41 (0.91) | 0 (0.00) | 0 (0.00) | <5 (<7.50) | <5 (<2.50) | 97 (4.10) | 142 (1.99) |
| **Ethnicity** |  |  |  |  |  |  |  |
| White | 4410 (97.72) | 43 (100.00) | 31 (100.00) | 25 (92.59) | 135 (88.82) | 2293 (97.00) | 6937 (97.29) |
| Non-white | 103 (2.28) | 0 (0.00) | 0 (0.00) | <5 (<7.50) | 17 (11.18) | 71 (3.00) | 193 (2.71) |
| **Income quintile (BU net worth)** |  |  |  |  |  |  |  |
| Lower quantile | 665 (14.74) | 6 (13.95) | 6 (19.35) | 8 (29.63) | 39 (25.66) | 440 (18.61) | 1164 (16.33) |
| Second quantile | 835 (18.50) | 8 (18.60) | 8 (25.81) | 5 (18.52) | 35 (23.03) | 511 (21.62) | 1402 (19.66) |
| Third quantile | 938 (20.78) | 8 (18.60) | <5 (<15.00) | 9 (33.33) | 38 (25.00) | 478 (20.22) | 1475 (20.69) |
| Fourth quantile | 1016 (22.51) | 7 (16.28) | 7 (22.58) | 5 (18.52) | 25 (16.45) | 470 (19.88) | 1530 (21.46) |
| Highest quantile | 1037 (22.98) | 14 (32.56) | 6 (19.35) | 0 (0.00) | 15 (9.87) | 434 (18.36) | 1506 (21.12) |
| Missing | 22 (0.49) | 0 (0.00) | 0 (0.00) | 0 (0.00) | 0 (0.00) | 31 (1.31) | 53 (0.74) |
| **Income quintile (BU equiv income)** |  |  |  |  |  |  |  |
| Lower quantile | 776 (17.19) | 7 (16.28) | 7 (22.58) | 6 (22.22) | 44 (28.95) | 507 (21.45) | 1347 (18.89) |
| Second quantile | 851 (18.86) | 5 (11.63) | 5 (16.13) | <5 (<15.00) | 43 (28.29) | 529 (22.38) | 1437 (20.15) |
| Third quantile | 907 (20.10) | 5 (11.63) | <5 (<15.00) | 9 (33.33) | 27 (17.76) | 483 (20.43) | 1435 (20.13) |
| Fourth quantile | 982 (21.76) | 15 (34.88) | 7 (22.58) | 7 (25.93) | 22 (14.47) | 470 (19.88) | 1503 (21.08) |
| Highest quantile | 975 (21.60) | 11 (25.58) | 8 (25.81) | <5 (<5.00) | 16 (10.53) | 344 (14.55) | 1355 (19.00) |
| Missing | 22 (0.49) | 0 (0.00) | 0 (0.00) | 0 (0.00) | 0 (0.00) | 31 (1.31) | 53 (0.74) |
| **Highest qualification** |  |  |  |  |  |  |  |
| Missing | 80 (1.77) | 0 (0.00) | <5 (<7.50) | 0 (0.00) | <5 (<2.50) | 10 (0.42) | 93 (1.30) |
| nvq4/nvq5/degree or equiv | 860 (19.06) | 10 (23.26) | 7 (22.58) | <5 (<15.00) | 6 (3.95) | 343 (14.51) | 1230 (17.25) |
| higher ed below degree | 654 (14.49) | 6 (13.95) | 5 (16.13) | 5 (18.52) | 8 (5.26) | 317 (13.41) | 995 (13.96) |
| nvq3/gce a level equiv | 421 (9.33) | 7 (16.28) | <5 (<10.00) | <5 (<15.00) | 7 (4.61) | 199 (8.42) | 640 (8.98) |
| nvq2/gce o level equiv | 901 (19.96) | 6 (13.95) | 5 (16.13) | <5 (<15.00) | 23 (15.13) | 448 (18.95) | 1387 (19.45) |
| nvq1/cse other grade equiv | 148 (3.28) | <5 (<2.50) | 0 (0.00) | <5 (<5.00) | 5 (3.29) | 104 (4.40) | 259 (3.63) |
| foreign/other | 658 (14.58) | 7 (16.28) | 6 (19.35) | <5 (<15.00) | 22 (14.47) | 283 (11.97) | 979 (13.73) |
| no qualification | 791 (17.53) | 6 (13.95) | <5 (<10.00) | 7 (25.93) | 80 (52.63) | 660 (27.92) | 1547 (21.70) |
| **Government Office Region** |  |  |  |  |  |  |  |
| North East | 319 (7.07) | <5 (<5.00) | <5 (<5.00) | <5 (<5.00) | 8 (5.26) | 100 (4.23) | 431 (6.04) |
| North West | 503 (11.15) | <5 (<7.50) | <5 (<5.00) | <5 (<7.50) | 19 (12.50) | 283 (11.97) | 811 (11.37) |
| Yorkshire and the Humber | 442 (9.79) | <5 (<7.50) | <5 (<5.00) | <5 (<15.00) | 16 (10.53) | 292 (12.35) | 758 (10.63) |
| East Midlands | 500 (11.08) | <5 (<5.00) | 6 (19.35) | <5 (<15.00) | 25 (16.45) | 249 (10.53) | 785 (11.01) |
| West Midlands | 480 (10.64) | 5 (11.63) | 5 (16.13) | <5 (<15.00) | 19 (12.50) | 233 (9.86) | 745 (10.45) |
| East of England | 611 (13.54) | <5 (<10.00) | 7 (22.58) | <5 (<15.00) | 18 (11.84) | 309 (13.07) | 952 (13.35) |
| London | 341 (7.56) | 13 (30.23) | <5 (<5.00) | <5 (<15.00) | 12 (7.89) | 201 (8.50) | 572 (8.02) |
| South East | 772 (17.11) | 5 (11.63) | 6 (19.35) | <5 (<15.00) | 24 (15.79) | 414 (17.51) | 1225 (17.18) |
| South West | 531 (11.77) | 7 (16.28) | <5 (<10.00) | <5 (<7.50) | 11 (7.24) | 275 (11.63) | 829 (11.63) |
| Missing | 14 (0.31) | 0 (0.00) | 0 (0.00) | 0 (0.00) | 0 (0.00) | 8 (0.34) | 22 (0.31) |

## Table S12: Demographic characteristics by sexual attraction in older adults

|  | **Entirely with opposite** | **Mostly w. opp., but some experience w. same** | **Equally with opposite and same** | **Mostly w. same, but some experience w. opp.** | **Entirely with same** | **No sexual experience in lifetime** | **Missing** | **Total** |
| --- | --- | --- | --- | --- | --- | --- | --- | --- |
| N= | 5666 | 218 | 57 | 19 | 46 | 87 | 1037 | 7130 |
| **Sex** |  |  |  |  |  |  |  |  |
| Male | 2586 (45.64) | 71 (32.57) | 15 (26.32) | 7 (36.84) | 28 (60.87) | 19 (21.84) | 458 (44.17) | 3184 (44.66) |
| Female | 3080 (54.36) | 147 (67.43) | 42 (73.68) | 12 (63.16) | 18 (39.13) | 68 (78.16) | 579 (55.83) | 3946 (55.34) |
| **Age bands** |  |  |  |  |  |  |  |  |
| 50-59 | 594 (10.48) | 43 (19.72) | <5 (<7.50) | <5 (<20.00) | 10 (21.74) | <5 (<5.00) | 260 (25.07) | 916 (12.85) |
| 60-69 | 2293 (40.47) | 101 (46.33) | 19 (33.33) | 8 (42.11) | 15 (32.61) | 20 (22.99) | 368 (35.49) | 2824 (39.61) |
| 70-79 | 1826 (32.23) | 60 (27.52) | 26 (45.61) | 6 (31.58) | 17 (36.96) | 31 (35.63) | 267 (25.75) | 2233 (31.32) |
| 80-89 | 832 (14.68) | 14 (6.42) | 9 (15.79) | <5 (<15.00) | <5 (<10.00) | 25 (28.74) | 129 (12.44) | 1015 (14.24) |
| 90+ | 121 (2.14) | 0 (0.00) | 0 (0.00) | 0 (0.00) | 0 (0.00) | 8 (9.20) | 13 (1.25) | 142 (1.99) |
| **Ethnicity** |  |  |  |  |  |  |  |  |
| White | 5536 (97.71) | 215 (98.62) | 55 (96.49) | 19 (100.00) | 46 (100.00) | 80 (91.95) | 986 (95.08) | 6937 (97.29) |
| Non-white | 130 (2.29) | <5 (<2.50) | <5 (<5.00) | 0 (0.00) | 0 (0.00) | 7 (8.05) | 51 (4.92) | 193 (2.71) |
| **Income quintile (BU net worth)** |  |  |  |  |  |  |  |  |
| Lower quantile | 864 (15.25) | 32 (14.68) | 12 (21.05) | <5 (<20.00) | 10 (21.74) | 30 (34.48) | 213 (20.54) | 1164 (16.33) |
| Second quantile | 1085 (19.15) | 43 (19.72) | 12 (21.05) | <5 (<15.00) | 5 (10.87) | 28 (32.18) | 227 (21.89) | 1402 (19.66) |
| Third quantile | 1187 (20.95) | 36 (16.51) | 19 (33.33) | <5 (<15.00) | 9 (19.57) | 19 (21.84) | 203 (19.58) | 1475 (20.69) |
| Fourth quantile | 1272 (22.45) | 51 (23.39) | 8 (14.04) | 6 (31.58) | 11 (23.91) | 8 (9.20) | 174 (16.78) | 1530 (21.46) |
| Highest quantile | 1217 (21.48) | 56 (25.69) | 6 (10.53) | 6 (31.58) | 11 (23.91) | <5 (<2.50) | 208 (20.06) | 1506 (21.12) |
| Missing | 41 (0.72) | 0 (0.00) | 0 (0.00) | 0 (0.00) | 0 (0.00) | 0 (0.00) | 12 (1.16) | 53 (0.74) |
| **Income quintile (BU equiv income)** |  |  |  |  |  |  |  |  |
| Lower quantile | 1030 (18.18) | 46 (21.10) | 17 (29.82) | <5 (<20.00) | 10 (21.74) | 34 (39.08) | 207 (19.96) | 1347 (18.89) |
| Second quantile | 1163 (20.53) | 31 (14.22) | 15 (26.32) | <5 (<20.00) | 7 (15.22) | 24 (27.59) | 194 (18.71) | 1437 (20.15) |
| Third quantile | 1155 (20.38) | 35 (16.06) | 10 (17.54) | <5 (<15.00) | 5 (10.87) | 17 (19.54) | 211 (20.35) | 1435 (20.13) |
| Fourth quantile | 1219 (21.51) | 44 (20.18) | 7 (12.28) | 5 (26.32) | 13 (28.26) | 8 (9.20) | 207 (19.96) | 1503 (21.08) |
| Highest quantile | 1058 (18.67) | 62 (28.44) | 8 (14.04) | 6 (31.58) | 11 (23.91) | <5 (<5.00) | 206 (19.86) | 1355 (19.00) |
| Missing | 41 (0.72) | 0 (0.00) | 0 (0.00) | 0 (0.00) | 0 (0.00) | 0 (0.00) | 12 (1.16) | 53 (0.74) |
| **Highest qualification** |  |  |  |  |  |  |  |  |
| Missing | 18 (0.32) | <5 (<0.50) | 0 (0.00) | <5 (<7.50) | 0 (0.00) | 0 (0.00) | 73 (7.04) | 93 (1.30) |
| nvq4/nvq5/degree or equiv | 998 (17.61) | 53 (24.31) | <5 (<7.50) | 8 (42.11) | 9 (19.57) | 8 (9.20) | 151 (14.56) | 1230 (17.25) |
| higher ed below degree | 818 (14.44) | 28 (12.84) | 13 (22.81) | <5 (<15.00) | 5 (10.87) | 9 (10.34) | 120 (11.57) | 995 (13.96) |
| nvq3/gce a level equiv | 504 (8.90) | 33 (15.14) | <5 (<7.50) | <5 (<7.50) | 6 (13.04) | <5 (<5.00) | 88 (8.49) | 640 (8.98) |
| nvq2/gce o level equiv | 1124 (19.84) | 39 (17.89) | 9 (15.79) | <5 (<7.50) | 7 (15.22) | 10 (11.49) | 197 (19.00) | 1387 (19.45) |
| nvq1/cse other grade equiv | 215 (3.79) | 8 (3.67) | <5 (<7.50) | 0 (0.00) | <5 (<5.00) | <5 (<5.00) | 28 (2.70) | 259 (3.63) |
| foreign/other | 783 (13.82) | 35 (16.06) | 8 (14.04) | <5 (<20.00) | 7 (15.22) | 9 (10.34) | 134 (12.92) | 979 (13.73) |
| no qualification | 1206 (21.28) | 21 (9.63) | 17 (29.82) | <5 (<20.00) | 10 (21.74) | 44 (50.57) | 246 (23.72) | 1547 (21.70) |
| **Government Office Region** |  |  |  |  |  |  |  |  |
| North East | 314 (5.54) | 11 (5.05) | <5 (<7.50) | 0 (0.00) | <5 (<5.00) | 5 (5.75) | 96 (9.26) | 431 (6.04) |
| North West | 634 (11.19) | 16 (7.34) | <5 (<7.50) | <5 (<25.00) | <5 (<10.00) | 9 (10.34) | 140 (13.50) | 811 (11.37) |
| Yorkshire and the Humber | 615 (10.85) | 24 (11.01) | 10 (17.54) | <5 (<7.50) | 6 (13.04) | 10 (11.49) | 92 (8.87) | 758 (10.63) |
| East Midlands | 631 (11.14) | 19 (8.72) | 6 (10.53) | 0 (0.00) | <5 (<10.00) | 11 (12.64) | 114 (10.99) | 785 (11.01) |
| West Midlands | 549 (9.69) | 28 (12.84) | 6 (10.53) | <5 (<15.00) | <5 (<10.00) | 9 (10.34) | 147 (14.18) | 745 (10.45) |
| East of England | 814 (14.37) | 28 (12.84) | 8 (14.04) | <5 (<25.00) | <5 (<5.00) | 10 (11.49) | 86 (8.29) | 952 (13.35) |
| London | 440 (7.77) | 24 (11.01) | 6 (10.53) | <5 (<15.00) | 11 (23.91) | 11 (12.64) | 78 (7.52) | 572 (8.02) |
| South East | 1011 (17.84) | 41 (18.81) | 8 (14.04) | <5 (<25.00) | <5 (<10.00) | 16 (18.39) | 141 (13.60) | 1225 (17.18) |
| South West | 640 (11.30) | 27 (12.39) | 6 (10.53) | <5 (<15.00) | 9 (19.57) | 6 (6.90) | 139 (13.40) | 829 (11.63) |
| Missing | 18 (0.32) | 0 (0.00) | 0 (0.00) | 0 (0.00) | 0 (0.00) | 0 (0.00) | <5 (<0.50) | 22 (0.31) |

## Table S13: Demographic characteristics by sexual experience in older adults

|  | **Entirely with opposite** | **Mostly w. opp., but some experience w. same** | **Equally with opposite and same** | **Mostly w. same, but some experience w. opp.** | **Entirely with same** | **No sexual experience in lifetime** | **Missing** | **Total** |
| --- | --- | --- | --- | --- | --- | --- | --- | --- |
| N= | 5790 | 158 | 23 | 27 | 33 | 79 | 1020 | 7130 |
| **Sex** |  |  |  |  |  |  |  |  |
| Male | 2574 (44.46) | 85 (53.80) | 6 (26.09) | 13 (48.15) | 20 (60.61) | 27 (34.18) | 459 (45.00) | 3184 (44.66) |
| Female | 3216 (55.54) | 73 (46.20) | 17 (73.91) | 14 (51.85) | 13 (39.39) | 52 (65.82) | 561 (55.00) | 3946 (55.34) |
| **Age bands** |  |  |  |  |  |  |  |  |
| 50-59 | 616 (10.64) | 24 (15.19) | <5 (<10.00) | 6 (22.22) | 8 (24.24) | <5 (<5.00) | 258 (25.29) | 916 (12.85) |
| 60-69 | 2319 (40.05) | 85 (53.80) | 9 (39.13) | 11 (40.74) | 11 (33.33) | 20 (25.32) | 369 (36.18) | 2824 (39.61) |
| 70-79 | 1878 (32.44) | 38 (24.05) | 8 (34.78) | 9 (33.33) | 12 (36.36) | 28 (35.44) | 260 (25.49) | 2233 (31.32) |
| 80-89 | 854 (14.75) | 11 (6.96) | <5 (<20.00) | <5 (<5.00) | <5 (<7.50) | 21 (26.58) | 122 (11.96) | 1015 (14.24) |
| 90+ | 123 (2.12) | 0 (0.00) | 0 (0.00) | 0 (0.00) | 0 (0.00) | 8 (10.13) | 11 (1.08) | 142 (1.99) |
| **Ethnicity** |  |  |  |  |  |  |  |  |
| White | 5658 (97.72) | 152 (96.20) | 22 (95.65) | 27 (100.00) | 33 (100.00) | 75 (94.94) | 970 (95.10) | 6937 (97.29) |
| Non-white | 132 (2.28) | 6 (3.80) | <5 (<5.00) | 0 (0.00) | 0 (0.00) | <5 (<7.50) | 50 (4.90) | 193 (2.71) |
| **Income quintile (BU net worth)** |  |  |  |  |  |  |  |  |
| Lower quantile | 887 (15.32) | 28 (17.72) | <5 (<15.00) | <5 (<15.00) | 5 (15.15) | 36 (45.57) | 201 (19.71) | 1164 (16.33) |
| Second quantile | 1127 (19.46) | 19 (12.03) | <5 (<15.00) | 5 (18.52) | 5 (15.15) | 17 (21.52) | 225 (22.06) | 1402 (19.66) |
| Third quantile | 1204 (20.79) | 33 (20.89) | 6 (26.09) | 5 (18.52) | <5 (<15.00) | 18 (22.78) | 205 (20.10) | 1475 (20.69) |
| Fourth quantile | 1292 (22.31) | 40 (25.32) | 6 (26.09) | 6 (22.22) | 9 (27.27) | 6 (7.59) | 171 (16.76) | 1530 (21.46) |
| Highest quantile | 1240 (21.42) | 37 (23.42) | <5 (<20.00) | 7 (25.93) | 10 (30.30) | <5 (<5.00) | 206 (20.20) | 1506 (21.12) |
| Missing | 40 (0.69) | <5 (<2.50) | 0 (0.00) | 0 (0.00) | 0 (0.00) | 0 (0.00) | 12 (1.18) | 53 (0.74) |
| **Income quintile (BU equiv income)** |  |  |  |  |  |  |  |  |
| Lower quantile | 1070 (18.48) | 33 (20.89) | 6 (26.09) | 5 (18.52) | 7 (21.21) | 29 (36.71) | 197 (19.31) | 1347 (18.89) |
| Second quantile | 1188 (20.52) | 24 (15.19) | 6 (26.09) | <5 (<15.00) | 5 (15.15) | 18 (22.78) | 193 (18.92) | 1437 (20.15) |
| Third quantile | 1173 (20.26) | 26 (16.46) | <5 (<20.00) | <5 (<15.00) | <5 (<7.50) | 20 (25.32) | 206 (20.20) | 1435 (20.13) |
| Fourth quantile | 1239 (21.40) | 30 (18.99) | <5 (<10.00) | 7 (25.93) | 9 (27.27) | 8 (10.13) | 208 (20.39) | 1503 (21.08) |
| Highest quantile | 1080 (18.65) | 44 (27.85) | 5 (21.74) | 8 (29.63) | 10 (30.30) | <5 (<7.50) | 204 (20.00) | 1355 (19.00) |
| Missing | 40 (0.69) | <5 (<2.50) | 0 (0.00) | 0 (0.00) | 0 (0.00) | 0 (0.00) | 12 (1.18) | 53 (0.74) |
| **Highest qualification** |  |  |  |  |  |  |  |  |
| Missing | 18 (0.31) | <5 (<2.50) | 0 (0.00) | <5 (<5.00) | 0 (0.00) | 0 (0.00) | 73 (7.16) | 93 (1.30) |
| nvq4/nvq5/degree or equiv | 1001 (17.29) | 49 (31.01) | <5 (<15.00) | 10 (37.04) | 8 (24.24) | 10 (12.66) | 149 (14.61) | 1230 (17.25) |
| higher ed below degree | 833 (14.39) | 23 (14.56) | 5 (21.74) | 5 (18.52) | <5 (<5.00) | 7 (8.86) | 121 (11.86) | 995 (13.96) |
| nvq3/gce a level equiv | 516 (8.91) | 26 (16.46) | <5 (<10.00) | <5 (<7.50) | <5 (<10.00) | <5 (<5.00) | 88 (8.63) | 640 (8.98) |
| nvq2/gce o level equiv | 1148 (19.83) | 27 (17.09) | <5 (<15.00) | <5 (<5.00) | 7 (21.21) | 7 (8.86) | 194 (19.02) | 1387 (19.45) |
| nvq1/cse other grade equiv | 227 (3.92) | 0 (0.00) | 0 (0.00) | 0 (0.00) | <5 (<7.50) | <5 (<5.00) | 28 (2.75) | 259 (3.63) |
| foreign/other | 813 (14.04) | 19 (12.03) | <5 (<15.00) | <5 (<15.00) | 5 (15.15) | 5 (6.33) | 130 (12.75) | 979 (13.73) |
| no qualification | 1234 (21.31) | 13 (8.23) | 7 (30.43) | <5 (<15.00) | 7 (21.21) | 45 (56.96) | 237 (23.24) | 1547 (21.70) |
| **Government Office Region** |  |  |  |  |  |  |  |  |
| North East | 322 (5.56) | 5 (3.16) | <5 (<5.00) | 0 (0.00) | <5 (<7.50) | 7 (8.86) | 94 (9.22) | 431 (6.04) |
| North West | 639 (11.04) | 16 (10.13) | <5 (<15.00) | <5 (<7.50) | <5 (<15.00) | 10 (12.66) | 137 (13.43) | 811 (11.37) |
| Yorkshire and the Humber | 635 (10.97) | 15 (9.49) | <5 (<20.00) | 5 (18.52) | 0 (0.00) | 11 (13.92) | 88 (8.63) | 758 (10.63) |
| East Midlands | 646 (11.16) | 12 (7.59) | 0 (0.00) | 0 (0.00) | <5 (<10.00) | 8 (10.13) | 116 (11.37) | 785 (11.01) |
| West Midlands | 565 (9.76) | 17 (10.76) | <5 (<10.00) | <5 (<7.50) | <5 (<15.00) | 8 (10.13) | 147 (14.41) | 745 (10.45) |
| East of England | 830 (14.34) | 24 (15.19) | <5 (<10.00) | <5 (<15.00) | <5 (<7.50) | 7 (8.86) | 84 (8.24) | 952 (13.35) |
| London | 449 (7.75) | 21 (13.29) | <5 (<10.00) | 8 (29.63) | 6 (18.18) | 11 (13.92) | 75 (7.35) | 572 (8.02) |
| South East | 1032 (17.82) | 30 (18.99) | 5 (21.74) | <5 (<15.00) | 6 (18.18) | 11 (13.92) | 138 (13.53) | 1225 (17.18) |
| South West | 654 (11.30) | 18 (11.39) | <5 (<20.00) | <5 (<15.00) | 6 (18.18) | 6 (7.59) | 137 (13.43) | 829 (11.63) |
| Missing | 18 (0.31) | 0 (0.00) | 0 (0.00) | 0 (0.00) | 0 (0.00) | 0 (0.00) | <5 (<0.50) | 22 (0.31) |

## Table S14: Health outcomes by sexual identity in adolescents

|  | **Completely heterosexual** | **Mainly heterosexual** | **Bisexual** | **Mainly Gay or Lesbian** | **Completely Gay or Lesbian** | **Other** | **Do Not Know** | **Prefer Not to Say** | **Total** |
| --- | --- | --- | --- | --- | --- | --- | --- | --- | --- |
|  | N= 7,888 | N= 1,101 | N= 656 | N= 90 | N= 160 | N= 157 | N= 16 | N = 35 | N= 10,103 |
| **On the whole, I am satisfied with myself** |  |  |  |  |  |  |  |  |  |
| Strongly agree | 1787  (22.65) | 124  (11.26) | 51  (7.77) | 7  (7.78) | 29  (18.13) | 14  (8.92) | 0  (0) | 8  (22.86) | 2020  (19.99) |
| Agree | 4506  (57.12) | 579  (52.59) | 301  (45.88) | 43  (47.78) | 59  (36.88) | 65  (41.40) | 11  (68.75) | 14  (40.00) | 5578  (55.21) |
| Disagree | 1181  (14.97) | 304  (27.61) | 227  (34.60) | 24  (26.67) | 50  (31.25) | 42  (26.75) | <5 (<20.00) | 5  (14.29) | 1836  (18.17) |
| Strongly disagree | 401  (5.08) | 92  (8.36) | 76  (11.59) | 16  (17.78) | 22  (13.75) | 33  (21.02) | <5  (<7.50) | <5  (<7.50) | 643  (6.36) |
| Do Not Know | 5  (0.06) | <5  (<0.50) | <5  (<0.50) | 0  (0) | 0  (0) | <5  (<2.50) | <5  (<7.50) | <5  (<7.50) | 14  (0.14) |
| I do not wish to answer | 8  (0.10) | 0  (0) | 0  (0) | 0  (0) | 0  (0) | 0  (0) | 0  (0) | <5  (<15.00) | 12  (0.12) |
| **How would you describe your health generally?** |  |  |  |  |  |  |  |  |  |
| Excellent | 2395  (30.36) | 265  (24.07) | 118  (17.99) | 15  (16.67) | 34  (21.25) | 27  (17.20) | <5  (<7.50) | 11  (31.43) | 2866  (28.37) |
| Very good | 3,124  (39.60) | 427  (38.78) | 237  (36.13) | 36  (40.00) | 5  (34.38) | 47  (29.94) | <5  (<15.00) | 9  (25.71) | 3937  (38.97) |
| Good | 1822  (23.10) | 293  (26.61) | 215  (32.77) | 26  (28.89) | 42  (26.25) | 59  (37.58) | 7  (43.75) | 10  (28.57) | 2474  (24.49) |
| Fair | 391  (4.96) | 85  (7.72) | 58  (8.84) | 11  (12.22) | 19  (11.88) | 18  (11.46) | 5  (31.25) | <5  (<10.00) | 590  (5.84) |
| Poor | 77  (0.95) | 17  (1.54) | 20  (3.05) | <5  (<2.50) | <5  (<2.50) | 5  (3.18) | 0  (0) | 0  (0) | 122  (1.21) |
| No answer | 81  (1.03) | 14  (1.27) | 8  (1.22) | 0  (0.00) | 7  (4.38) | <5  (<2.50) | <5  (<7.50) | <5  (<7.50) | 114  (1.13) |
| **KESSLER** |  |  |  |  |  |  |  |  |  |
|  | N=7880 | N=1100 | N=656 | N=90 | N=160 | N=157 | N=16 | N=28 | N=10,092 |
| Mean KESSLER (95% CI) | 6.50  (6.40, 6.60) | 9.36  (9.08, 9.64) | 11.18  (10.79, 11.56) | 10.79  (9.73, 11.85) | 10.09  (9.17, 11.01) | 10.78  (9.80, 11.76) | 7.75  (5.23, 10.27) | 5.36  (3.49, 7.22) | 7.28  (7.18, 7.37) |

Table S15: Health outcomes by sexual attraction in adolescents

|  | **Only to opposite** | **More often to opposite** | **About equally** | **More often to same** | **Only to same** | **Never sexually attracted** | **Do not know** | **I do not wish to answer** | **No answer** | **Total** |
| --- | --- | --- | --- | --- | --- | --- | --- | --- | --- | --- |
|  | N=7,656 | N=1,382 | N=385 | N=194 | N=137 | N=291 | N=11 | N=46 | N=1 | N=10,103 |
| **On the whole, I am satisfied with myself** | | | | | | | | | |  |
| Strongly agree | 1734 (22.65) | 150 (10.85) | 24 (6.23) | 14 (7.22) | 26 (18.98) | 57 (19.59) | <5 (<30.00) | 12 (26.09) | - | 2020 (19.99) |
| Agree | 4412 (57.63) | 664 (48.05) | 180 (46.75) | 92 (47.42) | 54 (39.42) | 155 (53.26) | <5 (<30.00) | 18 (39.13) | - | 5580 (55.21) |
| Disagree | 1127 (14.72) | 422 (30.54) | 130 (33.77) | 53 (27.32) | 40 (29.20) | 54 (18.56) | <5 (<30.00) | 7 (15.22) | - | 1836 (18.17) |
| Strongly disagree | 373 (4.87) | 143 (10.35) | 50 (12.99) | 35 (18.04) | 17 (12.41) | 21 (7.22) | 0 (0.00) | <5 (<7.50) | - | 643 (6.36) |
| Do Not Know | 5 (0.07) | <5 (<0.50) | <5 (<0.50) | 0 (0.00) | 0 (0.00) | <5 (<2.50) | <5 (<20.00) | <5 (<2.50) | - | 14 (0.14) |
| I do not wish to answer | 5 (0.07) | <5 (<0.50) | 0 (0.00) | 0 (0.00) | 0 (0.00) | <5 (<0.50) | 0 (0.00) | 5 (10.87) | - | 12 (0.12) |
| **How would you describe your health generally?** | | | | | | | | | |  |
| Excellent | 2340 (30.69) | 305 (22.07) | 68 (17.66) | 29 (14.95) | 33 (24.09) | 68 (23.37) | <5 (<2.50) | 12 (26.09) | - | 2,866 (28.37) |
| Very good | 3047 (39.80) | 509 (36.83) | 141 (36.62) | 78 (40.21) | 48 (33.04) | 97 (33.33) | <5 (<40.00) | 13 (28.26) | - | 3,937 (38.97) |
| Good | 1747 (22.82) | 395 (28.52) | 136 (35.32) | 58 (29.90) | 34 (24.82) | 86 (29.55) | <5 (<40.00) | 14 (30.43) | - | 2,474 (24.49) |
| Fair | 363 (4.74) | 126 (9.12) | 24 (6.23) | 21 (10.82) | 15 (10.95) | 32 (11.00) | <5 (<20.00) | 6 (13.04) | - | 590 (5.94) |
| Poor | 73 (0.95) | 27 (1.95) | 8 (2.08) | 5 (2.58) | <5 (<2.50) | 6 (2.06) | 0 (0.00) | <5 (<2.50) | - | 122 (1.21) |
| No answer | 76 (0.99) | 20 (1.45) | 8 (2.08) | <5 (<2.50) | 5 (3.65) | <5 (<2.50) | 0 (0.00) | 0 (0.00) | - | 144 (1.13 |
| **KESSLER** | | | | | | | | | |  |
|  | N=7652 | N=1382 | N=385 | N=194 | N=137 | N=290 | N=11 | N=35 | - | N=10092 |
| Mean KESSLER (95% CI) | 6.43 (6.33, 6.53) | 10.03 (9.78, 10.29) | 11.54 (11.03, 12.04) | 11.06 (10.32, 11.80) | 9.77 (8.79, 10.76) | 7.27 (6.63, 7.90) | 6.18 (2.51, 9.85) | 5.60 (3.91, 7.29) | - | 7.28 (7.18, 7.37) |

Table S16: Health outcomes by sexual identity in older adults

|  | **Heterosexual or Straight** | **Gay or Lesbian** | **Bisexual** | **Other** | **Prefer Not to Say** | **Missing** | **Total** |
| --- | --- | --- | --- | --- | --- | --- | --- |
| N= | 4513 | 43 | 31 | 27 | 152 | 2364 | 7130 |
| **Life Satisfaction** |  |  |  |  |  |  |  |
| Missing | 9 (0.20) | 0 (0.00) | 0 (0.00) | 0 (0.00) | 0 (0.00) | 46 (1.95) | 55 (0.77) |
| Strongly agree | 693 (15.36) | 7 (16.28) | 6 (19.35) | 8 (29.63) | 26 (17.11) | 329 (13.92) | 1069 (14.99) |
| Agree | 2221 (49.21) | 21 (48.84) | 13 (41.94) | 12 (44.44) | 70 (46.05) | 1004 (42.47) | 3341 (46.86) |
| Slightly agree | 710 (15.73) | <5 (<10.00) | 5 (16.13) | <5 (<15.00) | 21 (13.82) | 407 (17.22) | 1150 (16.13) |
| Neither agree nor disagree | 353 (7.82) | <5 (<10.00) | <5 (<15.00) | 0 (0.00) | 20 (13.16) | 211 (8.93) | 591 (8.29) |
| Slightly disagree | 283 (6.27) | <5 (<5.00) | <5 (<10.00) | <5 (<15.00) | 8 (5.26) | 159 (6.73) | 458 (6.42) |
| Disagree | 188 (4.17) | <5 (<10.00) | 0 (0.00) | <5 (<5.00) | 7 (4.61) | 135 (5.71) | 335 (4.70) |
| Strongly disagree | 56 (1.24) | <5 (<5.00) | 0 (0.00) | 0 (0.00) | 0 (0.00) | 73 (3.09) | 131 (1.84) |
| **Self-rated General Health** |  |  |  |  |  |  |  |
| Missing | 0 (0.00) | 0 (0.00) | 0 (0.00) | 0 (0.00) | 0 (0.00) | <5 (<0.50) | <5 (<0.50) |
| excellent | 566 (12.54) | 5 (11.63) | 6 (19.35) | <5 (<5.00) | 8 (5.26) | 187 (7.91) | 774 (10.86) |
| very good | 1362 (30.18) | 11 (25.58) | 6 (19.35) | 8 (29.63) | 30 (19.74) | 581 (24.58) | 1998 (28.02) |
| Good | 1487 (32.95) | 13 (30.23) | 12 (38.71) | 6 (22.22) | 48 (31.58) | 778 (32.91) | 2344 (32.88) |
| Fair | 797 (17.66) | 7 (16.28) | <5 (<10.00) | 7 (25.93) | 50 (32.89) | 522 (22.08) | 1386 (19.44) |
| Poor | 301 (6.67) | 7 (16.28) | 4 (12.90) | <5 (<15.00) | 16 (10.53) | 295 (12.48) | 627 (8.79) |
| **CES-D** |  |  |  |  |  |  |  |
| N= | 4513 | 43 | 31 | 27 | 152 | 2,357 | 7130 |
| **Mean CES-D (95% CI)** | 2.91 (2.87, 2.95) | 3.07 (2.65, 3.49) | 2.87 (2.45, 3.29) | 3.56 (2.96, 4.15) | 3.24 (3.01, 3.47) | 3.13 (3.08, 3.19) | 2.99 (2.96, 3.03) |

Table S17: Health outcomes by sexual attraction in older adults

|  | **Entirely with opposite** | **Mostly w. opp., but some experience w. same** | **Equally with opposite and same** | **Mostly w. same, but some experience w. opp.** | **Entirely with same** | **No sexual experience in lifetime** | **Missing** | **Total** |
| --- | --- | --- | --- | --- | --- | --- | --- | --- |
| N= | 5666 | 218 | 57 | 19 | 46 | 87 | 1037 | 7130 |
| **Life Satisfaction** |  |  |  |  |  |  |  |  |
| Missing | 43 (0.76) | 0 (0.00) | 0 (0.00) | 0 (0.00) | <5 (<2.50) | 1 (1.15) | 10 (0.96) | 55 (0.77) |
| Strongly agree | 849 (14.98) | 26 (11.93) | 11 (19.30) | 5 (26.32) | 11 (23.91) | 17 (19.54) | 150 (14.46) | 1069 (14.99) |
| Agree | 2640 (46.59) | 108 (49.54) | 28 (49.12) | 9 (47.37) | 21 (45.65) | 38 (43.68) | 497 (47.93) | 3341 (46.86) |
| Slightly agree | 931 (16.43) | 27 (12.39) | 5 (8.77) | <5 (<7.50) | <5 (<7.50) | 13 (14.94) | 170 (16.39) | 1150 (16.13) |
| Neither agree nor disagree | 478 (8.44) | 21 (9.63) | <5 (<7.50) | 0 (0.00) | <5 (<7.50) | <5 (<5.00) | 82 (7.91) | 591 (8.29) |
| Slightly disagree | 357 (6.30) | 21 (9.63) | 6 (10.53) | <5 (<7.50) | <5 (<5.00) | 8 (9.20) | 63 (6.08) | 458 (6.42) |
| Disagree | 264 (4.66) | 10 (4.59) | <5 (<7.50) | <5 (<20.00) | <5 (<5.00) | <5 (<5.00) | 50 (4.82) | 335 (4.70) |
| Strongly disagree | 104 (1.84) | 5 (2.29) | <5 (<2.50) | 0 (0.00) | <5 (<7.50) | <5 (<10.00) | 15 (1.45) | 131 (1.84) |
| **Self-rated General Health** |  |  |  |  |  |  |  |  |
| Missing | 0 (0.00) | <5 (<0.50) | 0 (0.00) | 0 (0.00) | 0 (0.00) | 0 (0.00) | 0 (0.00) | <5 (<0.50) |
| excellent | 603 (10.64) | 26 (11.93) | 6 (10.53) | 0 (0.00) | 9 (19.57) | 9 (10.34) | 121 (11.67) | 774 (10.86) |
| very good | 1605 (28.33) | 71 (32.57) | 9 (15.79) | 6 (31.58) | 10 (21.74) | 9 (10.34) | 288 (27.77) | 1998 (28.02) |
| Good | 1857 (32.77) | 72 (33.03) | 20 (35.09) | 10 (52.63) | 11 (23.91) | 25 (28.74) | 349 (33.65) | 2344 (32.88) |
| Fair | 1118 (19.73) | 35 (16.06) | 16 (28.07) | <5 (<15.00) | 8 (17.39) | 20 (22.99) | 187 (18.03) | 1386 (19.44) |
| Poor | 483 (8.52) | 13 (5.96) | 6 (10.53) | <5 (<7.50) | 8 (17.39) | 24 (27.59) | 92 (8.87) | 627 (8.79) |
| **CES-D** |  |  |  |  |  |  |  |  |
| N= | 5,659 | 218 | 57 | 19 | 46 | 87 | 1037 | 7,123 |
| **Mean CES-D (95% CI)** | 2.98 (2.94, 3.01) | 2.99 (2.81, 3.17) | 3.07 (2.70, 3.44) | 2.95 (2.40, 3.50) | 3.02 (2.61, 3.43) | 3.59 (3.24, 3.93) | 3.05 (2.96, 3.13) | 2.99 (2.96, 3.03) |

Table S18: Health outcomes by sexual experience in older adults

|  | **Entirely with opposite** | **Mostly w. opp., but some experience w. same** | **Equally with opposite and same** | **Mostly w. same, but some experience w. opp.** | **Entirely with same** | **No sexual experience in lifetime** | **Not answered** | **Total** |
| --- | --- | --- | --- | --- | --- | --- | --- | --- |
| N= | 5790 | 158 | 23 | 27 | 33 | 79 | 1020 | 7130 |
| **Life Satisfaction** |  |  |  |  |  |  |  |  |
| Missing | 41 (0.71) | 0 (0.00) | <5 (<10.00) | 0 (0.00) | <5 (<7.50) | <5 (<5.00) | 8 (0.78) | 55 (0.77) |
| Strongly agree | 870 (15.03) | 18 (11.39) | <5 (<15.00) | 6 (22.22) | 6 (18.18) | 21 (26.58) | 145 (14.22) | 1069 (14.99) |
| Agree | 2702 (46.67) | 83 (52.53) | 11 (47.83) | 13 (48.15) | 17 (51.52) | 25 (31.65) | 490 (48.04) | 3341 (46.86) |
| Slightly agree | 944 (16.30) | 22 (13.92) | <5 (<10.00) | <5 (<10.00) | <5 (<7.50) | 9 (11.39) | 169 (16.57) | 1150 (16.13) |
| Neither agree nor disagree | 488 (8.43) | 8 (5.06) | <5 (<10.00) | 0 (0.00) | <5 (<7.50) | 10 (12.66) | 81 (7.94) | 591 (8.29) |
| Slightly disagree | 375 (6.48) | 10 (6.33) | <5 (<15.00) | <5 (<5.00) | 0 (0.00) | 7 (8.86) | 62 (6.08) | 458 (6.42) |
| Disagree | 269 (4.65) | 9 (5.70) | 0 (0.00) | 5 (18.52) | <5 (<5.00) | <5 (<2.50) | 50 (4.90) | 335 (4.70) |
| Strongly disagree | 101 (1.74) | 8 (5.06) | 0 (0.00) | 0 (0.00) | <5 (<10.00) | <5 (<7.50) | 15 (1.47) | 131 (1.84) |
| **Self-rated General Health** |  |  |  |  |  |  |  |  |
| Missing | 0 (0.00) | <5 (<2.50) | 0 (0.00) | 0 (0.00) | 0 (0.00) | 0 (0.00) | 0 (0.00) | <5 (<0.50) |
| excellent | 610 (10.54) | 24 (15.19) | <5 (<5.00) | <5 (<15.00) | 7 (21.21) | 8 (10.13) | 121 (11.86) | 774 (10.86) |
| very good | 1643 (28.38) | 39 (24.68) | <5 (<20.00) | 9 (33.33) | 5 (15.15) | 12 (15.19) | 286 (28.04) | 1998 (28.02) |
| Good | 1896 (32.75) | 57 (36.08) | 11 (47.83) | 8 (29.63) | 10 (30.30) | 20 (25.32) | 342 (33.53) | 2344 (32.88) |
| Fair | 1147 (19.81) | 23 (14.56) | 6 (26.09) | <5 (<15.00) | <5 (<15.00) | 19 (24.05) | 183 (17.94) | 1386 (19.44) |
| Poor | 494 (8.53) | 14 (8.86) | <5 (<5.00) | <5 (<15.00) | 7 (21.21) | 20 (25.32) | 88 (8.63) | 627 (8.79) |
| **CES-D** |  |  |  |  |  |  |  |  |
| N= | 5783 | 158 | 23 | 27 | 33 | 79 | 1020 | 7123 |
| **Mean CES-D (95% CI)** | 2.98 (2.95, 3.02) | 2.86 (2.66, 3.06) | 3.30 (2.66, 3.95) | 2.52 (2.17, 2.87) | 3.03 (2.54, 3.52) | 3.48 (3.12, 3.84) | 3.05 (2.97, 3.14) | 2.99 (2.96, 3.03) |

Table S19: Health outcomes by sexual identity recoding strategy in older adults

|  | Heterosexual | SM | Total | Heterosexual | SM | Total |
| --- | --- | --- | --- | --- | --- | --- |
| **Recoding strategy** | **Restricted** | | | **Broad** | | |
| N= | 4513 | 74 | 4587 | 4513 | 253 | 4766 |
| **Life Satisfaction** |  |  |  |  |  |  |
| Agree  Odds Ratio (95%CI) | 1 | 0.76 (0.45, 1.30) |  | 1 | 0.84 (0.62, 1.14) |  |
| **Self-rated General Health** |  |  |  |  |  |  |
| Excellent or Very good  Odds Ratio (95%CI) | 1 | 0.81 (0.51, 1.31) |  | 1 | 0.56 (0.44, 0.76) |  |
| **CES-D** |  |  |  |  |  |  |
| **Mean CES-D (95% CI)** | 2.91 (2.87, 2.95) | 2.99 (2.69, 3.29) | 2.91 (2.87, 2.95) | 2.91 (2.87, 2.95) | 3.20 (3.02, 3.37) | 2.93 (2.89, 2.96) |

(ref: heterosexual)

Table S20: Health outcomes by sexual attraction recoding strategy in older adults

|  | Heterosexual | SM | Total | Heterosexual | SM | Total |
| --- | --- | --- | --- | --- | --- | --- |
| **Recoding strategy** | **Restricted** | | | **Broad** | | |
| N= | 5884 | 122 | 6006 | 5666 | 427 | 6093 |
| **Life Satisfaction** |  |  |  |  |  |  |
| Agree  Odds Ratio (95%CI) | 1 | 0.95 (0.62, 1.46) | - | 1 | 0.88 (0.70, 1.10) | - |
| **Self-rated General Health** |  |  |  |  |  |  |
| Excellent or Very good  Odds Ratio (95%CI) | 1 | 0.76 (0.52, 1.11) | - | 1 | 0.89 (0.73, 1.09) | - |
| **CES-D** |  |  |  |  |  |  |
| **Mean CES-D (95% CI)** | 2.98 (2.94, 3.01) | 3.03 (2.79, 3.28) | 2.98 (2.94, 3.01) | 2.98 (2.94, 3.01) | 3.12 (2.98, 3.26) | 2.99 (2.95, 3.02) |

(ref: heterosexual)

Table S21: Health outcomes by sexual experience recoding strategy in older adults

|  | Heterosexual | SM | Total | Heterosexual | SM | Total |
| --- | --- | --- | --- | --- | --- | --- |
| **Recoding strategy** | **Restricted** | | | **Broad** | | |
| N= | 5948 | 83 | 6031 | 5790 | 320 | 6110 |
| **Life Satisfaction** |  |  |  |  |  |  |
| **Agree**  **Odds Ratio (95%CI)** | 1 | 0.83 (0.51, 1.37) | - | 1 | 0.85 (0.65, 1.10) | - |
| **Self-rated General Health** |  |  |  |  |  |  |
| Excellent or Very good  Odds Ratio (95%CI) | 1 | 0.84 (0.53, 1.33) | - | 1 | 0.85 (0.67, 1.07) | - |
| **CES-D** |  |  |  |  |  |  |
| **Mean CES-D (95% CI)** | 2.98 (2.94, 3.01) | 2.94 (2.65, 3.23) | 2.91 (2.87, 2.95) | 2.98 (2.95, 3.02) | 3.03 (2.88, 3.19) | 2.98 (2.95, 3.02) |

(ref: heterosexual)

Table S22: Health outcomes by combined dimension recoding strategy in older adults

|  | Heterosexual | SM | Total | Heterosexual | SM | Total |
| --- | --- | --- | --- | --- | --- | --- |
| **Recoding strategy** | **Restricted** | | | **Broad** | | |
| N= | 6944 | 84 | 7028 | 6426 | 704 | 7130 |
| **Life Satisfaction** |  |  |  |  |  |  |
| Agree  Odds Ratio (95%CI) | 1 | 0.79 (0.49, 1.29) | - | 1 | 0.85 (0.71, 1.01) | - |
| **Self-rated General Health** |  |  |  |  |  |  |
| Excellent or Very good  Odds Ratio (95%CI) | 1 | 0.74 (0.46, 1.17) | - | 1 | 0.80 (0.67, 0.93) | - |
| **CES-D** |  |  |  |  |  |  |
| **Mean CES-D (95% CI)** | 2.99 (2.96, 3.02) | 3.10 (2.78, 3.41) | 2.99 (2.96, 3.02) | 2.98 (2.95, 3.01) | 3.14 (3.04, 3.25) | 2.99 (2.96, 3.03) |

(ref: heterosexual)

Table S23: Health outcomes by sexual identity recoding strategy in adolescents

|  | Heterosexual | SM | Total | Heterosexual | SM | Total |
| --- | --- | --- | --- | --- | --- | --- |
| **Recoding strategy** | **Restricted** | | | **Broad** | | |
| N= | 8989 | 906 | 9895 | 7888 | 2215 | 10103 |
| **Life Satisfaction** |  |  |  |  |  |  |
| Agree  Odds Ratio (95%CI) | 1 | 0.34 (0.29, 0.39) | - | 1 | 0.36 (0.33, 0.40) | - |
| **Self-rated General Health** |  |  |  |  |  |  |
| Excellent or Very good  Odds Ratio (95%CI) | 1 | 0.54 (0.47, 0.62) | - | 1 | 0.59 (0.54, 0.65) | - |
| **CES-D** |  |  |  |  |  |  |
| **Mean CES-D (95% CI)** | 6.85 (6.75, 6.94) | 10.95 (10.61, 11.29) | 7.22 (7.13, 7.32) | 6.50 (6.40, 6.60) | 10.05 (9.84, 10.27) | 7.27 (7.18, 7.37) |

(ref: heterosexual)

Table S23: Health outcomes by sexual attraction recoding strategy in adolescents

|  | Heterosexual | SM | Total | Heterosexual | SM | Total |
| --- | --- | --- | --- | --- | --- | --- |
| **Recoding strategy** | **Restricted** | | | **Broad** | | |
| N= | 9038 | 716 | 9754 | 7656 | 2446 | 10102 |
| **Life Satisfaction** |  |  |  |  |  |  |
| Agree  Odds Ratio (95%CI) | 1 | 0.36 (0.31, 0.42) | - | 1 | 0.36 (0.33, 0.40) | - |
| **Self-rated General Health** |  |  |  |  |  |  |
| Excellent or Very good  Odds Ratio (95%CI) | 1 | 0.57 (0.49, 0.66) | - | 1 | 0.57 (0.52, 0.62) | - |
| **CES-D** |  |  |  |  |  |  |
| **Mean CES-D (95% CI)** | 6.98 (6.88, 7.08) | 11.07 (10.68, 11.46) | 7.28 (7.18, 7.38) | 6.43 (6.33, 6.53) | 9.93 (9.72, 10.13) | 7.27 (7.18, 7.37) |

(ref: heterosexual)

Table S24: Health outcomes by combined dimension recoding strategy in adolescents

|  | Heterosexual | SM | Total | Heterosexual | SM | Total |
| --- | --- | --- | --- | --- | --- | --- |
| **Recoding strategy** | **Restricted** | | | **Broad** | | |
| N= | 9291 | 480 | 9771 | 7335 | 2746 | 10081 |
| **Life Satisfaction** |  |  |  |  |  |  |
| Agree  Odds Ratio (95%CI) | 1 | 0.35 (0.29, 0.42) | - | 1 | 0.37 (0.34, 0.41) | - |
| **Self-rated General Health** |  |  |  |  |  |  |
| Excellent or Very good  Odds Ratio (95%CI) | 1 | 0.57 (0.47, 0.68) | - | 1 | 0.58 (0.53, 0.63) | - |
| **CES-D** |  |  |  |  |  |  |
| **Mean CES-D (95% CI)** | 6.97 (6.87, 7.07) | 11.32 (10.88, 11.76) | 7.18 (7.09, 7.28) | 6.35 (6.25, 6.46) | 9.76 (9.56, 9.95) | 7.28 (7.18, 7.37) |

(ref: heterosexual)
